# Discovery of Anthocyanin Acyltransferase1 (AAT1) in Maize Using Genotyping-by-Sequencing (GBS)

**DOI:** 10.1534/g3.118.200630

**Published:** 2018-09-26

**Authors:** Michael N. Paulsmeyer, Patrick J. Brown, John A. Juvik

**Affiliations:** * Department of Crop Sciences, University of Illinois at Urbana-Champaign, Urbana, Illinois, 61801; † Department of Plant Sciences, University of California at Davis, Davis, California, 95616

**Keywords:** Acyltransferase, intensifier1, Acylation anthocyanins

## Abstract

The reduced acylation phenotype describes the inability of certain accessions of maize (*Zea mays* [*L*.]) to produce significant amounts of acylated anthocyanins, which are typically the most abundant pigments. Acylated anthocyanins are important for their association with stability and are therefore important for the various industries using anthocyanins as natural colorants to replace synthetic dyes. Many anthocyanin acyltransferases have been characterized in other species; however, no anthocyanin acyltransferases have been characterized in maize. Therefore, a mapping population was developed from a cross between mutant stock 707G and wild-type acylation line B73 to identify the locus associated with the reduced acylation trait. High-performance liquid chromatography was used to assay the pigment content and composition of 129 F_2_ lines generated in the mapping population. Recessive alleles of *Colorless1*, *Colored1*, and the reduced acylation mutant all decreased anthocyanin content while *Intensifier1* increased anthocyanin content in aleurone tissue. The association of increased proportions of acylation with increased anthocyanin content indicates acylation may be important for increasing the stability of anthocyanins *in vivo*. Genotyping-by-sequencing was used to create SNP markers to map the reduced acylation locus. In the QTL analysis, a segment of Chromosome 1 containing transferase family protein GRMZM2G387394 was found to be significant. A UniformMu *Mu* transposon knockout of GRMZM2G387394 demonstrated this gene has anthocyanidin malonyltransferase activity and will therefore be named *Anthocyanin Acyltransferase1* (*AAT1*). *AAT1* is the first anthocyanin acyltransferase characterized in a monocot species and will increase our knowledge of all acyltransferase family members.

Anthocyanins are the colorful molecules responsible for most of the red, blue, pink, and purple colors exhibited in plants. Anthocyanins are a diverse class of secondary metabolites with over 635 unique compounds discovered to date (He and Giusti 2010). The anthocyanin biosynthetic pathway is the most thoroughly studied secondary metabolite pathway in the plant kingdom (Irani *et al.* 2003). In maize, all the essential genes in the anthocyanin biosynthetic pathway have been cloned and sequenced to date, with *Purple aleurone1* completing the core pathway (Sharma *et al.* 2011). However, the genes involved with increasing the diversity of anthocyanin compounds in maize have not all been discovered.

The most common modification to anthocyanins in maize is acylation. Acylation is the esterification of organic acids, usually coumaroyl or malonic acid, to the glycoside of an anthocyanin molecule (Zhao *et al.* 2017). Most interest in acylated pigments is for their role in stability. Acylation has been shown to increase the stability of the anthocyanin molecule under heat stress, intense light, and high pH under processing conditions (Zhao *et al.* 2017). *In vivo*, acylation sterically hinders enzymatic breakdown and nucleophilic attack under cytosolic and vacuolar conditions (Suzuki *et al.* 2002; Bakowska-Barczak 2005). Addition of acyl groups to anthocyanins directly increases anthocyanin content (AC) by enhancing anthocyanin solubility and increasing uptake into the vacuoles (Suzuki *et al.* 2002; Zhao *et al.* 2011). Acylated anthocyanins may also have a beneficial role in health promotion. In a study analyzing the effect of blue maize extracts on various cancer cell lines, certain acylated anthocyanins showed strong correlations with antiproliferation (Urias-Lugo *et al.* 2015). Overall, increasing proportions of acylated anthocyanins in plant extracts would benefit human health and benefit the industries seeking to use anthocyanins as natural alternatives to synthetic dyes like FD&C Red 40.

Maize pigment extracts are an abundant source of acylated anthocyanins. The proportions of these compounds is usually greater than half of the total anthocyanins according to a survey of pigmented maize accessions (Paulsmeyer *et al.* 2017). Some pigmented accessions in the survey, however, exhibited a unique phenotype referred to as “reduced acylation” where acylated anthocyanins were drastically reduced or missing. It is hypothesized that this phenotype may be involved with the anthocyanin acyltransferase (AAT) synthesizing these compounds (Paulsmeyer *et al.* 2017). The diverse family of AATs belong to the BAHD superfamily of acyltransferases that acylate many diverse secondary metabolites (D’Auria 2006). Fortunately, several AATs have been characterized to date in other plant species (Unno *et al.* 2007). Specificity of AAT function is limited to particular acyl group substrates and sites of acylation. In maize, the acyl group substrate is most often malonic acid. The primary site of acylation in maize is at the 6’’-position of the 3-glucoside. The secondary site of acylation occurs on the 3′’-position to form various anthocyanidin 3-*O*-(3′’,6’’-dimalonyl) glucosides. Only one enzyme with the capability to produce these compounds has been characterized and sequenced: *Dendranthema × morifolium* (*Chrysanthemun morifolium*) anthocyanidin 3-*O*-3′’,6’’-*O*-dimalonyltransferase (Dm3MaT2) (Suzuki *et al.* 2004). Candidate AATs in maize should have some homology to Dm3MaT, although sequence identity among BAHD members can be as low as 25–34% (St-Pierre and De Luca 2000).

To map the locus associated with the reduced acylation phenotype, a mapping population was created by crossing B73, the maize reference line with normal acyltransferase function, to reduced acylation genetic stock 707G from the Maize Genetics Cooperation Stock Center (MGCSC; Urbana, IL, USA). High-performance liquid chromatography (HPLC) was used to phenotype the AC and composition in the 129 F_2_ progeny generated from this cross. The same individuals were genotyped using a double restriction enzyme digest modification of genotyping-by-sequencing (GBS) to generate SNPs. Genotyping-by-sequencing is a high-throughput SNP genotyping protocol that reduces the complexity of the genome by only sequencing fragments associated with restriction enzyme cut sites (Elshire *et al.* 2011). The protocol allows for the use methylation-sensitive restriction enzymes for enrichment of euchromatic regions and allows for hundreds of samples to be multiplexed in one sequencing run with the ligation of unique barcodes (Elshire *et al.* 2011). Presented here is a demonstration of how GBS can be effectively used to discover candidate genes even with small mapping populations and highly multiplexed individuals. The discovery of the AAT in this study, which will be designated *Anthocyanin Acyltransferase1* (*AAT1*), helps expand our knowledge of the anthocyanin biosynthetic pathway in maize and also our knowledge of AATs in general.

## Materials and Methods

### Plant Materials

Most reduced acylation mutants used in this study were first discovered in Paulsmeyer *et al.* (2017), but the list was expanded to include newly found mutant accessions M142X and M741I from the MGCSC, and Apache Red Cob from Siskiyou Seeds (Williams, OR, USA). UniformMu stock UfMu-09775 was donated by the MGCSC and was generated in a *Mutator* (*Mu*) transposon mutagenesis study (McCarty *et al.* 2005). Genetic stock 707G was also donated by the MGCSC and was chosen as the reduced acylation mutant parent for the F_2_ mapping population. B73 was chosen as the normal acylation parent, since the genome has been published for this line (Youens-Clark *et al.* 2011). All genetic stocks are available upon request by the corresponding author. Genetic stock 707G contributed many of the alleles for genes that influenced pigment production in the population. Photos of these traits can be seen in Figure S1. *Pericarp color1* (*P1*) was responsible for the red/white cob phenotype. *P1-ww* with white cobs was contributed by 707G, while functional allele *P1-wr* with red cobs was contributed by B73 (Grotewold *et al.* 1994). Genetic stock 707G contains a recessive *Intensifier1* gene known to increase AC in aleurone tissues when recessive (Burr *et al.* 1996). The stock also donated functional copies of two transcription factors that are required for aleurone pigmentation: *Colorless1* (*C1*) and *Colored1* (*R1*) (Andorf *et al.* 2010). 707G was selfed for three generations before crossing to B73, meaning it was not fully inbred (M. Sachs, personal communication, August 1, 2018). The cross between B73 and 707G was made in a winter nursery and pigmented F_2_ kernels were grown the next summer with 7.62 m plots spaced 0.76 m apart at the University of Illinois Vegetable Research Farm (40° 04′ 38.89″ N, 88° 14′ 26.18″ W). These plants were selfed to generate F_2:3_ kernels for analysis. Harvested ears were dried in a forced air dryer at ∼38° for five days before further analyses.

### HPLC Analyses

Approximately 50 F_2:3_ colored kernels of each F_2_ family were ground with a coffee grinder into a fine powder. Samples containing less than 50 kernels had approximately half of their colored kernels sampled. Following the extraction procedure from Paulsmeyer *et al.* (2017), a 2.0 g subsample of corn powder was added to 10 mL ACS reagent grade 2% (*v/v*) formic acid within a 15 mL centrifuge tube. The mixture was extracted overnight in the dark on an Innova 4000 incubator shaker (New Brunswick Scientific, Edison, NJ, USA) set at room temperature and 200 RPM. Extracts were filtered through a 25 mm 0.45 µm Millex Millipore (EMD Millipore, Merck KGaA, Darmstadt, Germany) LCR PTFE Syringe Filter after centrifugation at approximately 7500 x *g* on a Beckman J2-21M floor centrifuge with a JA-20.1 rotor (Beckman Coulter, Inc, Brea, CA, USA). For the UniformMu cross analyses, single kernels (∼0.2 g) were ground and extracted overnight in 5 mL 2% (*v/v*) formic acid as above. A 20 µL aliquot of each extract was injected into a Hitachi L-7200 HPLC (Hitachi High Technologies, Inc. Schaumburg, IL, USA) equipped with a Grace Prevail (W. R. Grace & Co., Columbia, MD, USA) C_18_ 5 µm analytical column (250 mm × 4.6 mm) and a Hitachi L-7455 Diode Array Detector quantifying absorbances at 520 nm. The column was heated to a constant 30.0°. The mobile phase consisted of 2% formic acid and 100% HPLC grade acetonitrile at a flow rate of 1 mL/min in the following linear gradient: 15% acetonitrile at 0 min, 30% acetonitrile at 10 min, and 15% acetonitrile at 15 min. After each sample, the column was allowed to equilibrate with 15% acetonitrile for 10 min. Samples were all run twice and averaged.

### Identification of Anthocyanins

**Figure 1:**
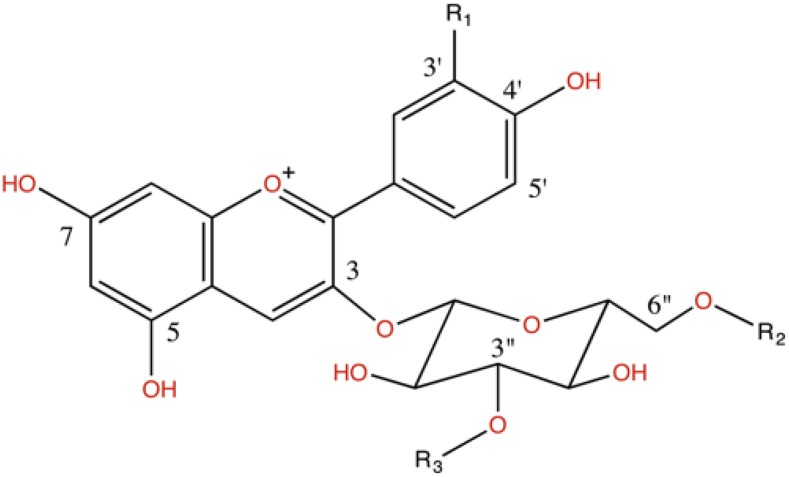
Structure of the most common anthocyanin molecules in maize. R1:H, R2:H, R3:H = Pelargonidin 3-Glucoside (Pg3G, ID #2) ; R1:OH, R2:H, R3:H = Cyanidin 3-Glucoside (C3G, ID #1); R1:CH_3_, R2:H, R3:H = Peonidin 3-Glucoside (Pn3G, ID #3); R1:H, R2:malonyl, R3:H = Pelargonidin 3-(6″-malonyl)glucoside (Pg3MG, ID #5); R1:OH, R2:malonyl, R3:H = Cyanidin 3-(6″-malonyl)glucoside (C3MG, ID #4); R1:CH_3_, R2:malonyl, R3:H = Peonidin 3-(6″-malonyl)glucoside (Pn3MG, ID #7*); R1:H, R2:malonyl, R3:malonyl = Pelargonidin 3-(3″,6″-dimalonyl)glucoside (Pg3DMG, ID #8); R1:OH, R2:malonyl, R3:malonyl = Cyanidin 3-(3″,6″-dimalonyl)glucoside (C3DMG, ID #7*); R1:CH_3_, R2:malonyl, R3:malonyl = Peonidin 3-(3″,6″-dimalonyl)glucoside (Pn3DMG, not detected). *Pigments co-elute and cannot be separated.

**Figure 2:**
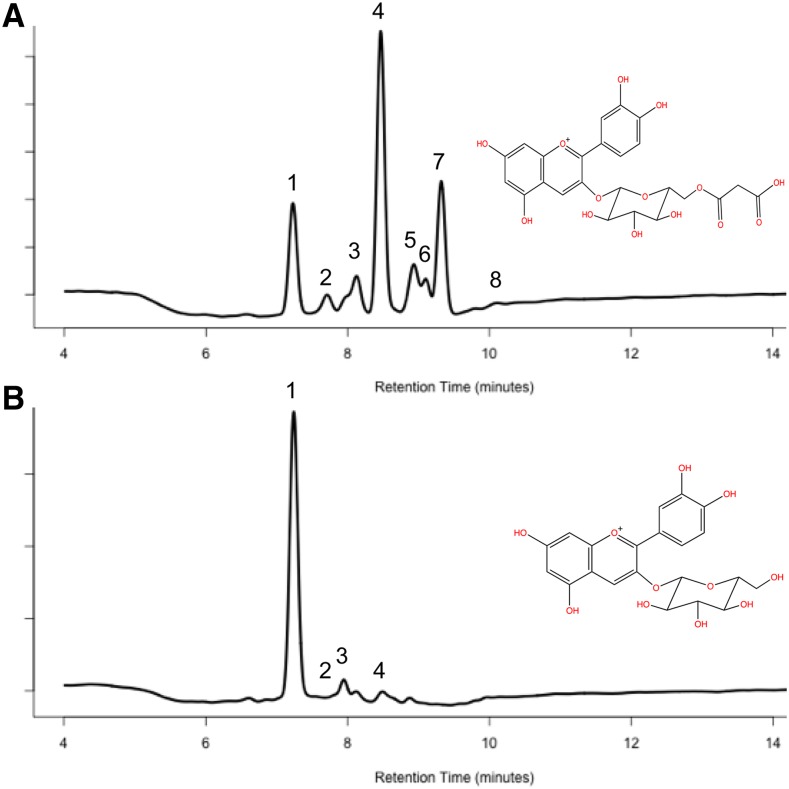
HPLC chromatograms of wild type and reduced acylation samples. A) Wild type profile with the structure of cyanidin 3-(6’’-malonyl) glucoside inset. B) Profile of a reduced acylation mutant with the structure of cyanidin 3-glucoside inset. *Note*: Peak labels correspond to the “ID #” listed in Figure 1.

Pigment identification was inferred from the method used in Paulsmeyer *et al.* (2017). A list of identified compounds in this study is in Figure 1. One limitation of this method is that several unknown compounds would co-elute with peonidin 3-glucoside (Pn3G; Figure 2). All peaks found under Pn3G would be summed together to simplify quantification. In addition, an unidentified peak arbitrarily labeled “ID #6” would appear next to pelargonidin 3-(6’’-malonyl) glucoside (Pg3MG; Figure 2). This peak was considered as a separate acylated anthocyanin compound because it eluted with other known acylated anthocyanins. This peak may be an isomer of an identified acylated anthocyanin.

### Phenotypic Evaluation

The Hitachi HPLC System Manager 4.0 software was used to integrate chromatogram peak areas. Phenotypes for individual anthocyanin compounds used these peaks areas as a measure. Anthocyanin content (AC) was quantified using the *Maíz Morado* external standard method in Paulsmeyer *et al.* (2017). The AC of Angelina’s Gourmet Maize Morado (Swanson, CT, USA) was determined to be 1000 mg/kg using cyanidin 3-glucoside (C3G) standard purchased from Phytolab GmbH & Co. (Vestenbergsgreuth, Germany) in a concentration gradient of 1 – 1000 µg/ml. The percentage of acylated anthocyanins of total anthocyanins was chosen as the phenotype to best describe reduced acylation mutants. Acylation percentage was calculated by summing peak areas of all acylated compounds in the sample, then dividing those by the total integrated area of the chromatogram and multiplying by 100. Alleles of *R1* were scored as 1/0 for the presence or absence of speckled kernels, respectively. The speckling phenotype is due to imprinting and forms when aleurone receives one *R1* allele from the pollen parent in a female *r1r1* endosperm (Kermicle 1970). The presence of recessive *C1* alleles was scored if the sample had yellow kernels without speckling, or if the proportion of colorless kernels per ear was approximately 50%.

### GBS Library Construction

Tissue from six to eight germinating F_2:3_ seedlings was collected into 1.2 mL microtiter tubes from the developing leaf whorl one week after planting. The six to eight F_2:3_ kernels used to represent F_2_ plants were chosen to emulate the sample from which they came in terms of pigmentation. A CTAB extraction protocol optimized for 96-well plates was used to isolate DNA. DNA quantification was completed using the PicoGreen assay (Molecular Probes, Eugene, OR). The GBS protocol outlined by Elshire *et al.* (2011) was used for library construction with a double restriction enzyme modification from Poland *et al.* (2012). Restriction enzymes chosen for library construction were *Hin*P1I and *Pst*I. In addition to the 129 samples from the reduced acylation mapping population, 201 other samples from an independent experiment were multiplexed during sequencing for 330 total samples. The independent population was for a separate mapping population not described here. Libraries were read in one sequencing lane on an Illumina HiSeq 2500 System (San Diego, CA, USA) at the Roy J. Carver Biotechnology Center at the University of Illinois in Urbana, IL, USA.

### SNP Discovery Pipeline

Genetic markers in the population were generated using the TASSEL 5.0 GBS v2 pipeline (Bradbury *et al.* 2007). Bowtie 2 (Langmead and Salzberg 2012) was used to align fragments to *Zea mays* B73 reference genome version 3 (Youens-Clark *et al.* 2011). Minor allele frequency (MAF) was set to 0.05. This resulted in 4,660,000 total SNPs for all 330 samples. Missing data ranged from 13.2 to 99.7% per sample. Samples with greater than 80% missing data were excluded. This changed the dataset to 328 samples, with one sample being lost from the reduced acylation population. The overall proportion of missing sites in the dataset was 36.32%. Next, insertion-deletions were removed and imported into Beagle 4.1 (Browning and Browning 2016). The options defined in Beagle 4.1 were a window length of 200 and a step size of 50. After imputation, the reduced acylation population was separated from the independent mapping population within TASSEL 5.0. A MAF of 0.05 was applied once again to the imputed SNPs resulting in a total of 8062 SNPs per sample for the reduced acylation population. Markers per chromosome ranged from 584 for Chromosome 10 to 1212 for Chromosome 1. Alleles were converted in TASSEL 5.0 to numerical genotypes 1 *vs.* 0 for major *vs.* minor or 0.5 for heterozygotes based on allele frequency in the population. Since 707G was a genetic stock and not completely inbred, some sites had three to four alleles, which were considered minor alleles. Numerical genotypes were multiplied by two to remove decimals and imported into R (R Core Team 2015) to perform QTL analyses.

### Statistical Analyses

To determine if genes of interest (*C1*, *In1*, *P1*, *R1*, and the reduced acylation mutant) were significantly altering the concentrations of individual pigments or total AC, ANOVAs were calculated using either Proc Reg or Proc Mixed in SAS 9.4 (SAS institute Inc., Cary, NC, USA) depending on the outputs needed. *C1*, *P1*, *R1*, and reduced acylation mutants were coded as 0 or 1 depending on if the sample contained dominant or homozygous recessive alleles, respectively, based on the phenotypes previously described. Genotypes of *In1* were inferred from alleles at the most significant site in the QTL analysis for log-transformed AC. All genes of interest were included in a linear model in Proc Reg to determine significance. Genes that were statistically significant were regressed to AC in Proc Reg to estimate the change in AC attributed by the significant genes. Variance components for the statistically significant genes of interest and the proportion of variation attributed by each gene were calculated in Proc Mixed with method equal to Type 3 using each gene as a random independent variable. All QTL analyses were run using the linear model (lm) function in R (R Core Team 2015) using phenotypes as the dependent and SNPs as the independent variable. A stepwise QTL analysis was used to find significant markers involved with the phenotype of interest. The first model run would assume a single QTL. The most significant marker from a single-QTL model would then be used as a covariate (**Q**) and the process repeated until no more markers were significant. The general model for stepwise QTL analysis is shown in Equation 1. The term $\overset{p}{\underset{k=2}{\sum }}\beta_{k}Q_{\mathrm{ki}}\;$represents the summation of the *k*th significant marker as a covariate for *p* covariates, while $\beta_{1}\mathrm{SN}P_{i}$ represents the numerical genotypes at a SNP site.

$$\mathrm{Equation}\;\text{1:}\;\mathrm{Phenotype}=\beta_{0}+\beta_{1}\mathrm{SN}P_{i}+\overset{p}{\underset{k=2}{\sum }}\beta_{k}Q_{\mathrm{ki}}+\varepsilon_{i}$$

The threshold for significant marker *p*-values in Equation 1 was determined by a Bonferroni correction of α = 0.05/*n* where *n* is the number of SNPs (*n* = 8062) and 0.05 is 95% confidence. All figures were generated in R (R Core Team 2015). All supplementary material, custom code, and datasets are available at https://figshare.com/projects/Discovery_of_Anthocyanin_Acyltransferase1_AAT1_in_maize_using_genotyping-by-sequencing_GBS_/38900.

### Data availability

The authors affirm that all data necessary for confirming the conclusions of this article are represented fully within the article and its tables and figures. Supplemental material available at Figshare: https://doi.org/10.25387/g3.7108748.

## Results

### Inheritance of the Trait

Ten accessions have been found to date that exhibit the reduced acylation trait. Pedigree analysis determined that all the reduced acylation MGCSC stocks are all derived from E. Coe’s *in1* genetic stocks developed in 1965 (E. Coe and P. Stinard, personal communication, August 1, 2018). The remaining stocks (50%) are all from diverse backgrounds suggesting that the mutation has occurred several times independently. The reduced acylation trait has no visual effect on the kernel phenotype, so selection for this trait is apparently indirect in these accessions. Reciprocal crosses of reduced acylation accessions to wild-type accessions returned normal acylation abundance meaning the trait is recessive to normal acylation. Several reduced acylation accessions were then crossed with each other to see if the trait was determined by the same loci in every accession. Accessions Ames 14276, Jerry Peterson Blue Dent, M142X, M741I, and X19EA failed to return normal acylation abundance when crossed to mapping population parent 707G indicating they are allelic.

### Summary of Mapping Population Phenotypes

**Figure 3:**
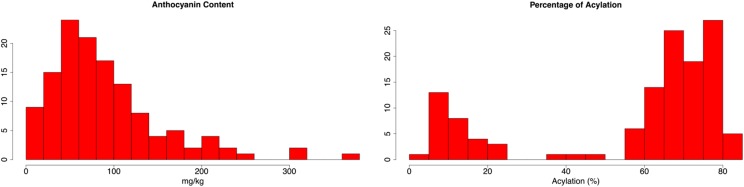
Histograms of two important phenotypes in the reduced acylation mapping population. (Left) anthocyanin content and (Right) percentage of acylation.

In the mapping population, 129 F_2_ individuals generated kernels for phenotyping and sequencing. Prior to sequencing, the reduced acylation mutants in the population were defined visually based on trends in the histogram for percentage of acylation (Figure 3). A few samples were intermediate in acylation percentage but the cutoff for a reduced acylation mutant was defined as less than 50% acylation based on the bimodal distribution seen in the histogram. The summary of all visual phenotypes and inferred *In1* genotypes is in Table 1. There were 32 progeny in the population displaying the reduced acylation phenotype, which is exactly the number of recessive samples expected for a population segregating at a single, recessive locus. The overall percentage of acylation in the wild-type samples averaged 70.8% while the mutant samples maintained an average of 15.0%. The overall population mean for AC was 96.2 mg/kg. The distribution of AC was skewed right (Figure 3) indicating that *in1* had a profound affect on AC in this population. Since a new HPLC method was used in this study, the coefficient of variation (CV) was calculated to determine repeatability. Coefficient of variation was calculated by dividing the standard deviation of AC for the replicates by the sample mean. The overall average CV was 3.09% confirming the method is acceptably reproducible. To see which loci had an effect on AC, a linear regression was modeled using all loci as random, independent variables. All variables except *P1* were significant at *P* < 0.05. Parameter estimates from the model calculate that *in1* raises AC an estimated 105.4 mg/kg while reduced acylation, *r1*, and *c1* lower AC 23.3 mg/kg, 24.2 mg/kg, and 29.1 mg/kg, respectively. The lowest AC sample was 11.84 mg/kg in a *C1c1/R1r1/In1X* reduced acylation plant and the highest was 369.54 mg/kg in a *C1C1/R1R1/in1in1* wild-type plant.

**Table 1:** SUMMARY OF PHENOTYPES IN THE MAPPING POPULATION

	Allele	Reduced Acylation	*In1*	*R1*	*C1*	*P1*	Total
AC (mg/kg)	Dominant	103.40	71.17	112.70	107.52	98.66	**96.19**
	Recessive	74.35	178.80	83.92	87.23	89.30	
Acylation (%)	Dominant	70.79	56.56	58.28	55.32	57.12	**56.92**
	Recessive	15.02	58.27	55.98	58.26	56.37	
Recessive Count		32	30	75	72	34	129

### Identification of Candidate Anthocyanin Acyltransferases

Since the most likely candidate for an AAT in maize should have anthocyanidin dimalonyltransferase activity, the dimalonyltransferase from chrysanthemum, Dm3MaT2 (Suzuki *et al.* 2004), was used as a query to find candidate AATs in blastp. The blastp search found seventeen candidates in maize with 25% or greater identity and 15% or greater query coverage in the reference proteins database. The seventeen candidates were aligned in COBALT (Papadopoulos and Agarwala 2007) using defaults. The candidates all showed close homology to the three motifs that are present in acyltransferases: HXXXD (Motif 1), DFGWG (Motif 2), and NYFGNC (Motif 3) (Suzuki *et al.* 2002). Motif 3 is an AAT-specific motif. Protein sequences were filtered on whether they contained Motif 3 present in chrysanthemum’s three functional anthocyanin malonyltransferases: XYF/LGNC (Suzuki *et al.* 2004). This filter resulted in nine candidate sequences (Table 2). Among these candidates, four were annotated as predicted AATs. One was even annotated as an anthocyanidin 3-*O*-glucoside 6’’-*O*-malonyltransferase. The remaining five candidates were uncharacterized proteins or transferase family proteins.

**Table 2:** CANDIDATE AATs IN MAIZE AFTER FILTERING

Gene Annotation	Gene Identifier (B73 RefGen_v3)	Chr	Start Position	End Position
Transferase	GRMZM2G387394	1	300173138	300174772
Malonyl-CoA:anthocyanin 5-*O*-glucoside-6’’’-*O*-malonyltransferase-like	GRMZM2G341253	2	165106367	165108162
Anthocyanin 5-aromatic acyltransferase	GRMZM2G075513	4	170843898	170845760
Malonyl-CoA:anthocyanidin 5-*O*-glucoside-6’’’-*O*-malonyltransferase-like	GRMZM2G316787	4	174072855	174074677
HXXXD-type acyltransferase	GRMZM2G382785	5	50372211	50374054
HXXXD-type acyltransferase	GRMZM2G436404	6	19122956	19124662
Anthocyanidin 3-*O*-glucoside 6’’-*O*-acyltransferase-like	GRMZM2G095340	6	72337117	72341167
Transferase family protein	GRMZM5G800407	10	142328397	142330129

Candidates were filtered based on homology to Dm3MaT2. Sequences had to have greater than 25% identity, greater than 15% coverage, and amino acid motif XYF/LGNC.

### QTL Analyses of Mapping Population Phenotypes

**Figure 4:**
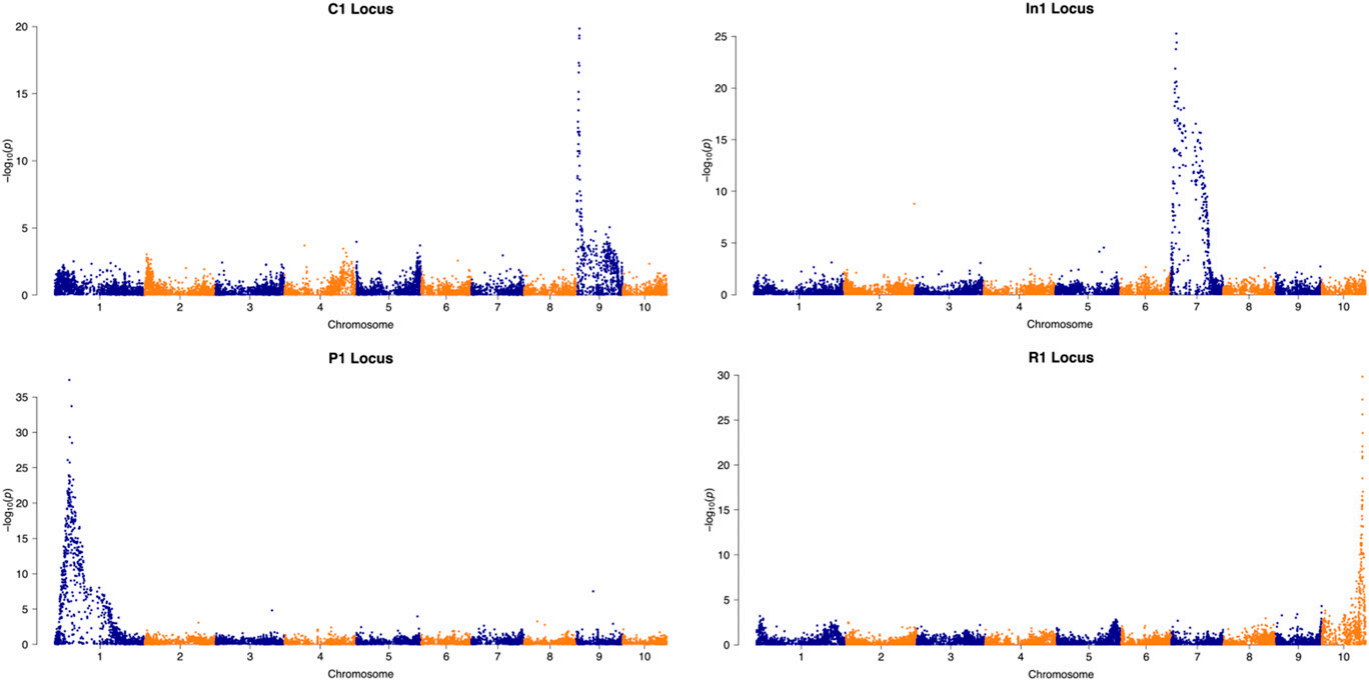
Manhattan plots for known genes segregating in the mapping population. All major QTL correspond to the known location of each gene (Andorf *et al.* 2010).

The GBS library constructed from the 129 individuals of this mapping population resulted in 128 genotypes with 8062 SNPs per genotype. SNPs were more prevalent in areas near the ends of chromosomes, but generally, the entire genome was represented. The average distance between SNPs in the final dataset was 255 Kb. Distance between SNPs under 1 Mb apart accounted for 95% of the dataset while 17.8% of those were under 1 Kb apart. Several control traits were mapped to assess the accuracy and robustness of the SNP dataset before the reduced acylation trait was mapped. The control loci—*C1* (Paz-Ares *et al.* 1987), *In1* (Burr *et al.* 1996), *P1* (Grotewold *et al.* 1994), and *R1* (Dellaporta *et al.* 1988)—segregating in the population have been previously mapped, sequenced, and characterized. The log-transformation of AC was used as the phenotype to map *In1*, since the distribution was skewed (Figure 3). Figure 4 shows the Manhattan plots generated from the four control traits. The most significant SNP correlations always corresponded to the known location of each gene. The most significant marker for *In1*, with a –log10 *p*-value of 21.36, was only 134 bp away from coding sequence. The most significant SNPs for loci *P1*, *C1*, and *R1* were 642 Kb, 1.7 Mb, and 637 Kb away, respectively (Andorf *et al.* 2010).

**Figure 5:**
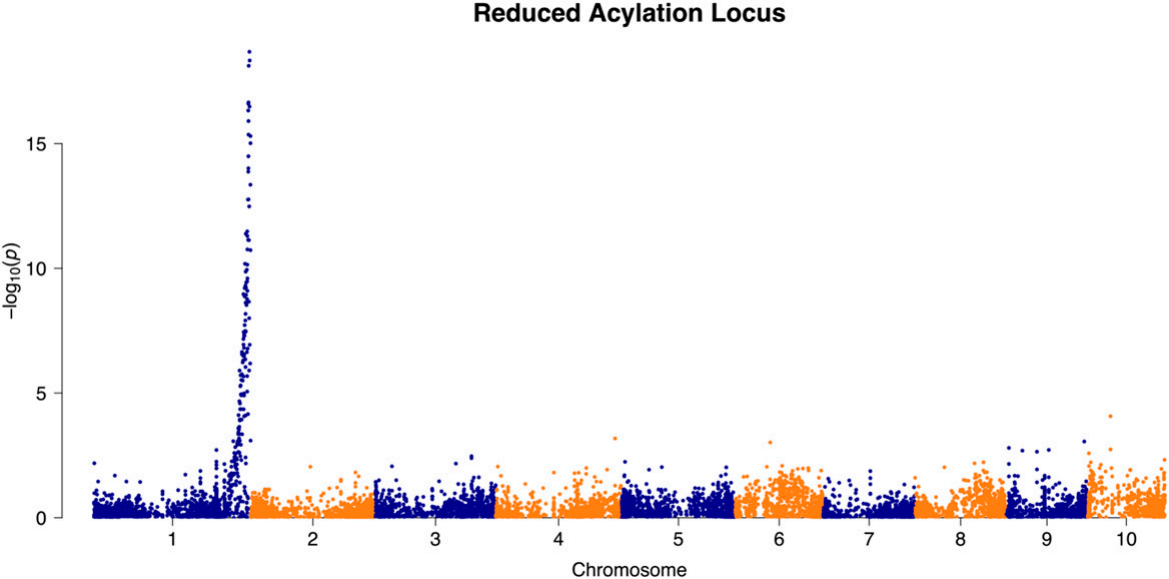
Reduced acylation locus Manhattan plot.

The control genes validated the robustness of the genotype dataset so that the reduced acylation phenotype could be mapped with high confidence. The phenotype chosen to represent the reduced acylation trait was percentage of acylation, since proportions of compounds were not affected by *in1*. The most significant marker with a –log10 *p*-value of 18.7 was at position 298,042,277 of Chromosome 1 (Figure 5). The Bonferroni correction established a 22.3 Mb interval of significant markers from positions 279,188,074 to 301,476,924 (the end of Chromosome 1). The significant genomic region for the reduced acylation trait spanned only one of the candidate genes from Table 2. The candidate transferase gene designated GRMZM2G387394 in B73 reference genome version 3 is at position 300,173,138 of Chromosome 1 (Figure 5).

**Figure 6:**
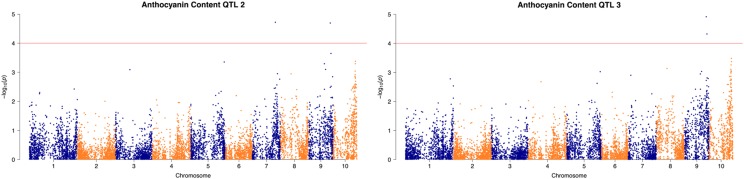
Manhattan plots for step-wise QTL analysis of anthocyanin content. *Left*:anthocyanin content QTL with *in1* as a covariate and a *P* < 1×10-4 significance threshold. *Right*: anthocyanin content QTL with *in1* and S7_144238875 covariates and a *P* < 1×10-4 significance threshold.

Pigment concentration of the grain is a trait breeders developing high anthocyanin-yielding varieties are seeking to improve. To find genomic regions associated with AC other than the transcription factors known, a model was fit with *In1* genotypes as a covariate and both log-transformed AC and raw AC as responses using Equation 1. No significant markers could be distinguished from both models at the significance threshold established by the highly conservative Bonferroni correction, so the significance threshold was relaxed to *P <* 1×10^−4^ in accordance with Li *et al.* (2016). With untransformed AC, a SNP on Chromosome 7 was most significant at position 144,238,875 (Figure 6). Near this marker is *MYB152*, a transcription factor that has been shown to correlate with *PAL* expression (Agarwal *et al.* 2016). When both *In1* and the SNP on Chromosome 7 were considered, SNP position 137,169,800 on Chromosome 9 became significant (Figure 6). No obvious gene candidates surrounding this marker could be found at this time.

In addition to AC, QTL analyses were run on individual compounds distinguished by HPLC. All compounds besides Pn3G and pelargonidin 3-(3′’,6’’-dimalonyl) glucoside (Pg3DMG) had two significant QTL corresponding to *AAT1* and *In1* (Figure S2). Pn3G and Pg3DMG had one significant QTL at the site of *In1*. When Pn3G was modeled with *In1* as a covariate using Equation 1, SNP markers spanning *R1* became significant. Only one significant locus could be found with Pg3DMG. The inability to detect more significant regions was most likely because only 26 samples had detectable amounts of Pg3DMG.

Variance components for each AC-altering locus were estimated for all anthocyanin phenotypes to determine the phenotypic variation attributed to each loci (Table 3). The gene that explained the most variance in AC for this population was *In1* (64.7%), while *R1*, *C1*, and the reduced acylation trait accounted for 3.02%, 4.56%, and 2.67% of the total variance, respectively. In general, *In1* explained the highest proportion of variance for all compounds and ranged from 28.1 to 56.5%. The lowest proportion of variance that could be detected as a significant QTL was 5.55% for *R1* with Pn3G.

**Table 3:** PROPORTION OF VARIANCE ATTRIBUTED TO EACH CONTROL LOCI FOR EACH ANTHOCYANIN COMPOUND

Compounds	In1 (%)	C1 (%)	R1 (%)	Reduced Acylation (%)	Residual (%)
**C3G**	35.32 ***	2.20 *	0.53	37.96 ***	23.99
**C3MG**	36.91 ***	2.45 *	2.26 *	34.43 ***	23.94
**Peak ID #6**	40.42 ***	2.35 *	0.62	23.26 ***	33.36
**Pg3DMG**	28.08 ***	0.97	3.40 *	8.14 *	59.41
**Pg3G**	42.11 ***	1.82 *	1.24 *	29.97 ***	24.87
**Pg3MG**	40.20 ***	2.55 *	2.28 *	25.32 ***	29.65
**Pn3G**	56.47 ***	5.55 **	5.55 **	0.38	32.04
**Pn3MG**	31.93 ***	2.38 *	3.22 *	37.45 ***	25.02
**AC**	64.66 ***	4.56 **	3.02 *	2.67 *	25.09

*** *P* < 0.0001 significance, ** *P* < 0.001 significance, * *P* < 0.05 significance.

### QTL Simulation

The dataset was sampled and simulated to determine the lowest number of samples possible to confirm a QTL. The two traits assessed were the presence of speckled kernels (*R1* phenotype, Figure S1) and percentage of acylation. Speckled kernels were represented in 75 of the 128 genotyped samples, while 32 of the 128 genotyped samples were determined to have the reduced acylation trait. Subsamples of 20, 40, 60, 80, and 100 genotypes were selected at random from the dataset. Sampling was repeated 100 times for each subsample of genotypes. The ability to detect QTL was arbitrarily defined as the most significant SNP above the Bonferroni significance threshold (*P* < 6.203×10^−6^) being only 10 Mb away from the known locus. The summary of the simulation is presented in Table 4. For the *R1* locus, forty samples were sufficient to pinpoint *R1* 100% of the time within 10 Mb. The average distance of the most significant SNP from the *R1* locus for the forty samples was 1.62 Mb away. Of the 20-genotype simulations, 39% significantly pinpointed the locus within 10 Mb, while 36% were considered false positives. The average *p*-value for the significant markers ranged from a –log10 *p*-value of 10.72 to 63.47. The high *p*-values associated with the 20-genotype subsamples may be due to ascertainment bias. For percentage of acylation, the QTL could be detected 100% of the time with only sixty samples. The average distance from the candidate acyltransferase for sixty samples was 2.27 Mb. In 37% of the simulations, the reduced acylation QTL could be mapped to the correct location with only twenty genotypes. One simulation was able to establish significance with only three of twenty reduced acylation mutants. The average significance for percentage of acylation ranged from 6.57 to 15.15 –log10 *p*-value. The reduced acylation simulation had more false positives then the *R1* locus. For the 40-genotype and 20-genotype subset, 6% and 25% of the significant markers were considered false positives, respectively.

**Table 4:** QTL SIMULATION STUDY

Sample Size	Within 10 Mb*	False Positives	Avg. Proportion Recessive *	Min. Proportion Recessive *	Avg. *p*-value (–log10) *	Avg. Distance From Known Locus (bp) *
20	39%	36%	58.7%	40.00%	63.47	8,994,347
40	100%	0%	56.1%	37.50%	10.72	1,623,113
60	100%	0%	57.83%	46.67%	15.12	748,586
80	100%	0%	57.89%	51.25%	19.38	678,954
100	100%	0%	57.77%	53.00%	23.88	675,179

Sample Size	Within 10 Mb*	False Positives	Avg. Proportion Recessive *	Min. Proportion Recessive *	Avg. *p*-value (–log10) *	Avg. Distance From Known Locus (bp) *
20	37%	25%	27.8%	15.00%	6.57	5,703,808
40	85%	6%	24.9%	12.50%	7.80	2,790,210
60	100%	0%	24.7%	16.67%	10.30	2,269,732
80	100%	0%	25.2%	17.50%	12.49	2,111,612
100	100%	0%	25.3%	20.00%	15.15	2,177,236

* Only if significant with a Bonferroni correction at –log10(p) ≥ 5.2074 and in the right location (within 10 Mb)

### UniformMu Knockout of GRMZM2G387394

To determine if the candidate acyltransferase on Chromosome 1 was associated with the formation of acylated anthocyanins, a UniformMu stock (McCarty *et al.* 2005), designated UfMu-09775, with a *Mutator* (*Mu*) transposon insertion within GRMZM2G387394 was crossed to reduced acylation mutant 707G. UfMu-09775 was heterozygous for the insertion and would theoretically confer the mutant phenotype in 50% of the kernels crossed to 707G. In the cross, 14 of 25 F_1_ kernels exhibited the reduced acylation phenotype, which is indicative of the 50% ratio expected (χ^2^ test, H_0_ = 50%, *P* = 0.5485). Therefore, it can be concluded that GRMZM2G387394 is at least required for anthocyanidin 3-*O*-glucoside 6’’-*O*-malonyltransferase function in maize and is responsible for the reduced acylation phenotype. Given this knowledge, GRMZM2G387394 from now on will be designated *Anthocyanin Acyltransferase1* or *AAT1*.

### Phylogenetics of Acyltransferase Members

At least nineteen flavonoid acyltransferases have been characterized to date (Unno *et al.* 2007; Kim *et al.* 2009). All characterized flavonoid acyltransferases are from eudicot species with the exception of one flavonoid malonyltransferase in *Oryza sativa* (Kim *et al.* 2009). The bootstrap consensus neighbor-joining phylogenetic tree constructed in Mega7 of sixteen known flavonoid acyltransferases with the maize candidate acyltransferase is shown in Figure S3 (Felsenstein 1985; Kumar *et al*. 2016; Saito and Nei 1987). Monocots formed their own clade and the phylogenetic tree otherwise matched previously reported phylogenies (Unno *et al.* 2007). The monocot clade is more closely related to the other malonyltransferases than the 5-*O*-glucoside or aromatic AATs. Unno *et al.* (2007) demonstrated that Thr-35, Leu-37, Ala-38, and Gln-51 are all important acyl-acceptor binding sites in Dm3MaT2. In maize, AAT1 Leu-37 and Ala-38 are conserved, while Gln-51 is functionally similar to Ser-69 in AAT1. Thr-35 is not present in AAT1. Three additional amino acids in Dm3MaT2—Tyr-411, Ala-413, and Lys-419—were required for AAT function as well (Unno *et al.* 2007). All but Lys-419 had structurally similar counterparts in AAT1. Due to the low identity between AAT1 and Dm3MaT2, functional domains of AATs can be inferred and should be investigated for future studies.

## Discussion

The reduced acylation phenotype in maize is due to anthocyanin acyltransferase *AAT1* and had a significant effect on AC in the mapping population. This finding agrees with the hypothesis that aliphatic acylation has a role in protecting the anthocyanin molecule. Therefore, increasing the proportion of acylated anthocyanins could have a positive influence on increasing AC *in vivo*. In addition to the reduced acylation mutant allele of *aat1*, *In1*, *C1*, and *R1* all have a significant effect on AC. The quantifiable change in AC associated with alleles of each of these anthocyanin regulators has, to the best of our knowledge, never been studied. Here only pigmented kernels were sampled for *C1* and *R1*, which will bias the results toward higher AC. Despite this, significant differences could be found among the combinations of each gene regulator. The effect of *In1* may be inexact, given the genotypes of *In1* were inferred from low-coverage SNP sites where heterozygotes may have been miscalled. The high significance (–log10 *p*-value 21.36) and relative proximity (134 bp away from the transcription start site) of the most significant marker for log-transformed AC along with the approximate 1:3 Mendelian ratio of homozygous recessive *in1* genotypes to dominant *In1* genotypes provide evidence that inferred *In1* genotypes may be accurate.

GBS is a simple and robust method for reduced representation sequencing. The ability to multiplex 384 or more samples per sequencing run greatly reduces costs. In addition, all steps from DNA extraction to library constriction can be completed in 96-well plate format, which helps maximize efficiency. In this study, 330 samples from two independent mapping populations were analyzed in one sequencing lane. A problem with many highly multiplexed GBS datasets is the amount of missing and incomplete data due to low-coverage sequencing (Swarts *et al.* 2014). In the initial dataset with 330 samples, two samples were discarded that had missing data over 80%. These samples most likely failed in the PCR or ligation steps of GBS library preparation. The amount of missing data has implications on how imputation fills in SNPs. Despite the uncertainty in variant calling, imputation has been shown to increase power to detect QTL (Fragoso *et al.* 2016). Missing data not only has an effect of imputation but also on heterozygote calling. Phenotypes may not correlate with SNP markers even though flanking markers are highly significant. This issue is apparent in Manhattan plots. Log-transformed AC had the largest QTL and spanned 269 SNPs. Of those, only 155 SNPs were significant with the Bonferroni significance threshold. In contrast, ascertainment bias from low-coverage sequences resulted in some false positive associations for some control traits. The conservative Bonferroni threshold should control false positives, but even in *P1*, a single marker on a different chromosome was significant (Figure 4). Overall, the effect of missing data and incorrectly called bases is seemingly negligible for this study since the most significant SNP markers for each control loci aligned correctly and only a single significant region was found for the reduced acylation trait.

Numerous significant marker associations may be a problem if several candidate genes resided within a single QTL. In this study, the QTL for reduced acylation only spanned a single candidate, so the width was acceptable. In the future, a new mapping approach based on linkage between markers, like bin-mapping, may be necessary to establish thresholds to differentiate candidates and establish a narrower confidence interval (Chen *et al.* 2014). Recombination rate in this population is partially to blame for the large number of significant markers. With more recombination events, smaller areas in the genome will remain correlated to the trait due to the breakage of linkage blocks. More recombination events can be introduced with a greater population size. For example, in a study that analyzed F_2_ progeny for grain yield traits, the *R1* gene was also mapped as a control, but used 611 progeny. *R1* was fine-mapped to an approximately 700 Kb confidence interval, as opposed to the 27.0 Mb interval between significant markers presented here (Chen *et al.* 2014). In addition to increasing population size, intermating the F_2_ genotypes could have been a way to increase recombination events. This scheme was utilized to develop the intermated B73 x Mo17 (IBM) population in maize that is used extensively for mapping traits (Lee *et al.* 2002; Eichten *et al.* 2011). Intermating requires additional generations, which is one reason it was not utilized here.

Despite the low SNP coverage relative to other GBS studies, the mapping population was able to map traits with one to three loci. The simulation here showed that QTL could be confirmed with as little as forty to sixty samples depending on the proportion of the recessive allele (Table 4). The power to correctly detect QTL is best when the dominant/recessive phenotypes are in nearly even proportions. The high power of detection presented here is in direct contrast to a previous study that could only establish significance for a single-locus trait at a chromosome-wide scale with 91 samples (Heffelfinger *et al.* 2014).

For quantitative traits, the ability to detect multiple QTL is important. More than three QTL could not be found in this mapping population since *in1* and *aat1* explained most of the variability in most traits. The possible number of QTL all depends on the nature of the trait of interest. If the trait is highly heritable and controlled by many genes with smaller effects, more than three significant associations may be possible. For example, Chen *et al.* (2014) were able to distinguish seven QTL correlated with tassel branch number in a large population of 692 F_2_ plants. The proportion of variance explained by the QTL in this trait ranged from 2.0 to 6.3%, which was the typical proportion of variance of *R1* and *C1* in the reduced acylation population. Moreover, another study utilizing GBS for quantitative traits found 35 QTL for plant height, ear height, and internode number with phenotypic variances ranging from 2.6 to 15.68% with 314 recombinant inbred lines (Zhou *et al.* 2016). Plant height, ear height, and tassel branch number are highly heritable traits as anthocyanin composition is expected to be (Paulsmeyer *et al.* 2017). Future work aimed at increasing AC would have to expand the mapping population to confirm the presence of the smaller effect QTL found in this study (Figure 6).

### Conclusions

The anthocyanin acyltransferase in maize, *AAT1*, synthesizes the majority of acylated anthocyanins, which are the most common anthocyanin compounds in maize. As confirmed in this study, acylated anthocyanins are important for increasing AC *in vivo* and may be important for use in natural colorants or for human health. Results of this study also demonstrate the effectiveness of utilizing GBS for discovering new genes. GBS is a simple and reliable high-throughput protocol that allows for multiplexing of numerous samples. In this study, GBS was able to discover a new gene in maize involved with the production of acylated anthocyanins, even with a small mapping population size that was highly multiplexed in a single sequencing lane. Knockouts of *AAT1* generated by a *Mu* transposon insertion returned the reduced acylation phenotype. With this it could be concluded that *AAT1* is the gene responsible for the reduced acylation trait. Whether or not this gene has dimalonyltransferase activity is yet to be determined. Current work is being aimed at expressing recombinant protein and assaying enzyme activity. Overall, the discovery of *AAT1* increases our understanding of the family of AATs and fills in missing steps of the anthocyanin biosynthetic pathway in maize.
